# Perinatal Pharmacology

**DOI:** 10.1155/2014/101620

**Published:** 2014-04-13

**Authors:** Karel Allegaert, Vassilios Fanos, Johannes N. van den Anker, Stephanie Laër

**Affiliations:** ^1^Department of Development and Regeneration, KU Leuven and Neonatal Intensive Care Unit, University Hospitals Leuven, Herestraat 49, 3000 Leuven, Belgium; ^2^Department of Surgery, Section of Neonatal Intensive Care Unit, Puericulture Institute and Neonatal Section, University of Cagliari, Cagliari, Italy; ^3^Departments of Pediatrics, Pharmacology, Physiology, and Integrative Systems Biology, George Washington University School of Medicine and Health Sciences, Washington DC, USA; ^4^Intensive Care, Erasmus MC-Sophia Children's Hospital, Rotterdam, The Netherlands; ^5^Department of Pediatric Pharmacology, University Children's Hospital Basel, Basel, Switzerland; ^6^Department of Clinical Pharmacy and Pharmacotherapy, Heinrich Heine University of Düsseldorf, Universitätsstraße 1, 40225 Düsseldorf, Germany

Effective and safe drug administration should be based on knowledge that integrates the evolving physiological characteristics of the individual patient who will receive the drug with the pharmacokinetics (PK) and pharmacodynamics (PD) of the prescribed drug. Consequently, clinical pharmacology in neonates and pregnant women is as dynamic and diverse as the specific populations considered [1, 2]. Despite this diversity, there is currently little integration of available knowledge to guide and optimize pharmacotherapy in these populations [3–6].

Mothers are commonly exposed to drugs during pregnancy, at delivery, or in the postpartum period, and this includes women with preexisting comorbidities. Despite this, dosing regimens are still commonly extrapolated from regimens initially validated in adults and do not consider the pregnancy related changes in physiology nor issues related to fetal effects before or breastfeeding related exposure after delivery [1, 6]. The same holds true for neonates. The most obvious covariates in neonates relate to growth and development, reflected and quantified by birth weight, current weight, or age—either postnatal, gestational, or postmenstrual age. There is already at least one log order of variability in weight (<0.5 up to 5 kg) while both the height velocity rate (10–20 cm/year) and the increase in body weight (50% increase in the first 6 weeks) reflect the dynamics of a rapidly evolving biological system during perinatal life. The maturation related variability is further aggravated by interfering disease characteristics (e.g., renal failure, sepsis, and growth restriction) or treatment modalities (e.g., comedication, extracorporeal membrane oxygenation, and whole body cooling).

Since infants and pregnant women warrant a focused approach due to the physiological changes related to maturation (fetus, newborn) or pregnancy, understanding these changes to predict exposure/effects is necessary. Modelling emerged as a promising tool to improve prediction of exposure/effects [3, 4, 6]. However, these methods need further validation before this approach can be implemented. In addition, both effects and side effects may be population specific. This necessitates the validation of biomarkers (e.g., liver enzymes, renal biomarkers, and blood pressure) commonly applied in other populations or exploration to develop, evaluate, and validate new approaches to assess drug effect or side effects (PD) in infants or pregnant women that are valid and appropriate for clinical use. Finally, practices and clinical care also evolve. Mothers undergo surgical interventions during pregnancy to improve fetal outcome, while hypothermia to improve neurodevelopment outcome in term neonates or trends in respiratory support in preterm neonates affect pharmacotherapy during neonatal intensive care [1–6]. The different topics discussed in this special issue on perinatal pharmacology cover the broad field of perinatal pharmacology.

This includes animal experimental studies describing zonisamide pharmacokinetics in the pregnant rabbit and the impact of alfa-7 nicotinic receptor modulation on brain inflammation in a neonatal asphyxia model in mice. Such specific studies may support the subsequent development of new dosing and treatment strategies in human populations. This is of clinical relevance since both perinatal epileptic syndromes and perinatal asphyxia still suffer from poor long term outcome.

As mentioned earlier, off-label use of drugs is unfortunately common practice, even for population specific clinical syndromes like pregnancy related nausea or vomiting, i.c. hyperemesis of pregnancy, a common medical condition in pregnancy. There is an increasing trend to prescribe ondansetron, although the safety of ondansetron for use in human pregnancy has not been established. In the absence of prospective evaluation of such drugs, retrospective analysis of safety and tolerance data may provide caregivers with information on the presence and the extent of such adverse effects. This strategy and its limitations have been explored in a dataset on ondansetron exposure in 251 pregnant women from Western Australia. Off-label indications can also be explored on its effectiveness, as illustrated for prevention of preterm delivery following folic acid supplementation. It is well known that folic acid supplementation is recommended in the periconceptional period to prevent neural tube defects. Due to the involvement of folic acid in a number of cellular processes, other pregnancy outcomes such as miscarriage, recurrent miscarriage, low birth weight, preeclampsia, abruptio placentae, stillbirth, and preterm birth have also been investigated in an off-label setting. For the prevention of preterm birth, E. Mantovani et al. describe a discrepancy between the slight decreases in preterm birth in observational studies, a finding not consistent with the results of randomized controlled trials. At least these contrasting findings reillustrate the need to perform randomized controlled trials in this specific population.

During the study design and dose selection, population tailored pharmacokinetic modelling can be applied to predict the exposure/effect relationship, but these models need validation and optimization [3, 6]. The paper of J. G. C. van Hasselt et al. on cefazolin pharmacokinetics during pregnancy explores the feasibility to integrate the known physiological changes related to pregnancy (i.c. glomerular filtration rate, protein binding) to predict changes in cefazolin disposition during pregnancy. The authors compared a semiphysiological versus an empirical modelling in an attempt to further validate their semiphysiological model. Following validation, such semiphysiological models can subsequently be applied to other compounds that undergo the same elimination routes.

Two of the papers focus on aspects of neonatal clinical pharmacology. The integration of pharmacogenetics into perinatal pharmacology is complex, since it is related to ontogeny or phenotypic activity during pregnancy. To illustrate this, the impact of cytochrome P450 (CYP) 2D6 polymorphisms in part depends on the postmenstrual age at exposure [7]. Recently, a link between neonatal pharmacogenetics (i.c. *μ*-opioid receptor and catechol-*O*-methyltransferase single-nucleotide polymorphisms) and the presence and extent of neonatal abstinence syndrome following maternal opioid exposure has been documented [8]. In this special issue, the potential link between pharmacogenetics and the use of Serotonin Reuptake Inhibitors (SSRIs) during pregnancy and adverse neonatal outcome was explored. Finally, the impact of pharmacological strategies to facilitate endotracheal intubation following the introduction of the INSURE, that is, intubation-surfactant administration-extubation strategy in preterm neonates, is discussed. It is a nice illustration of the need to reconsider our pharmacological strategies once the outcome variables (i.c. shorter duration of analgosedation) change.

We are aware that the topics discussed are indeed very heterogeneous. This was however the primary aim of this special issue, since progress in perinatal pharmacology will only be possible when different types of research expertise, including but not limited to clinical researchers, (pharmaco) geneticists, PK/PD modellers, or researchers with an animal experimental background, collaborate with the common goal to improve pharmacotherapy in pregnant women and their infants. We aspire that this special issue will stimulate clinicians and clinician-scientists to collaborate as multidisciplinary teams to achieve this goal.
